# Supplementary material for: Are carbon emissions trading and green financial instruments synergistic? -Comprehensive quantitative research based on content analysis
Source: PLoS One. 2024 Mar 7;19(3):e0298601. doi: 10.1371/journal.pone.0298601 (PMC10919601; doi:10.1371/journal.pone.0298601)
Supplement: S1 Data — (ZIP) [file pone.0298601.s001.zip › Data/地级市Z_F工作报告.zip!/░▓╗╒/╬▀║■╩╨/└┤╫╘╣1⁄2╓┌║┼í╛┬φ┐╦╩2╛▌═°í┐_2014.docx]

——2014年1月14日在芜湖市第十五届人民代表大会第三次会议上

市长 潘朝晖

各位代表：

现在，我代表市人民政府，向大会报告政府工作，请予审议，并请市政协委员和其他列席人员提出意见。

一、经济社会发展保持良好势头

2013年，在中共芜湖市委的坚强领导下，我们按照稳中求进、稳中有为的总要求，科学谋划，主动作为，经济社会发展呈现出结构优化、质量提升、后劲增强、民生改善的良好局面。经济总量迈上新台阶，全市实现地区生产总值2090亿元（预计数，下同），比上年增长12%；财政收入381.7亿元，增长13.2%；全社会固定资产投资2040亿元，增长20%；社会消费品零售总额557亿元，增长14%；进出口总额54.3亿美元，增长19.1%；城镇新增就业8万人；城市居民人均可支配收入26160元，农民人均纯收入11078元，分别增长10%和14.5%。

一年来，我们主要做了以下工作：

（一）加快江北建设步伐。市委、市政府成立了江北新区建设指挥部，强力推进跨江发展。编制了江北新区概念规划和江北产业集中区总体规划，11座过江通道建设纳入国家规划。芜湖长江公路二桥、合福铁路长江公铁大桥公路桥接线、北沿江高速、江北和谐大道、无六路改造等重点项目开工建设，合福铁路、合裕线航道整治等工程加快推进，商合杭铁路获批立项。裕溪口水道上段公用航道开通。江北水厂、裕溪口天然气输配站建成运行。江北产业集中区成为省直接调度开发区的“排头兵”，50多公里路网、公用码头一期基本建成，起步区基本实现“九通一平”，开工建设各类项目47个，总投资规模303亿元。江北电子信息产业集聚区、海创新型节能材料等项目开工建设，宝能组团、江北总部经济和金融服务区、双钱轮胎二期等项目加快推进。无为经济开发区扩区新设城东园区获批。二坝、汤沟镇划入市区，鸠江区级机关移至江北办公。市级投入和融资担保16亿元，支持无为县、鸠江区、江北产业集中区建设。从2014年起，皖B牌号7座以下小客车及公交车芜湖长江大桥通行费实行财政全额补贴。

（二）促进经济健康较快增长。积极应对严峻复杂的经济环境，出台了促进经济持续健康较快发展的42条意见，实行“一企一组一策”，兑付奖补资金49.8亿元，落实减免税58亿元。取消、降低涉企收费60项，减轻负担3.6亿元。对困难企业减缴、缓缴社会保险费6.7亿元，发放稳岗和促进就业补贴1.2亿元。实行市级重大项目十天一调度、县区重点项目一月一调度。东旭高端显示器件装备、格力商用空调、大陆电子二期等项目开工建设，新兴铸管大型铸锻件、双汇食品、雅图视频、三花空调元器件、盾安制冷设备等项目竣工投产。充实了县区担保公司国有资本金，开展了产业与金融资本对接服务活动，缓解中小企业融资难。信义光能、海螺创业2家企业上市，宇业集团“借壳”上市。全市完成直接融资223亿元。

（三）推动产业扩张升级。坚持首位产业首位支持、支柱产业加速转型、战略性新兴产业重点培育。汽车及装备制造业实现产值2200亿元，增长14%。汽车及零部件、材料、电子电器、电线电缆四大支柱产业实现总产值3475亿元，增长15%。机器人、新型显示产业进入国家战略性新兴产业区域集聚发展试点。战略性新兴产业实现产值1190亿元，增长23%。奇瑞建立了新的研发体系和品牌体系，艾瑞泽7、瑞虎5、观致3和迪凯挖掘机等新产品陆续上市。工业机器人产业园、循环经济产业园、通用航空产业园、铸锻产业园开工建设，新型平板显示、文化科技、新材料、凯翼汽车、海创总部基地、新能源等产业园建设正在推进。编制了服务业集聚区规划、现代物流业发展规划。芜湖金融服务中心、伟星时代金融中心建成。各类股权投资企业认缴资金207亿元。社会融资总量4100亿元，较年初增加600亿元。南翔万商国际物流园初具规模。港口吞吐量突破9000万吨。文化创意产业园获批省级现代服务业集聚区。建立了首个省级广告产业园区。服务外包执行金额3.3亿美元，增长52%。新华联、利海等文化旅游项目开工。希尔顿、悦圆方、天泰等酒店开业。新增旅行社4家。方特主题公园接待游客390万人次，全市接待游客2340万人次，增长15%。成功举办了中国体育文化?体育旅游博览会、第二届中国（芜湖）电线电缆博览会、全国首个大学生专业电商创业大赛。淘宝网全省首家地市级馆“特色中国芜湖馆”运营。加快发展现代农业，50亩以上规模化土地流转面积达107万亩。新增开城、平铺2个省级现代农业示范区。省级以上产业化龙头企业达55家，实现规模农产品加工产值450亿元。

（四）增强科技创新能力。以汇聚创新要素、培育创新型企业、支持高科技创业活动为重点，为产业升级提供科技支撑。出台了打造经济升级版的意见、提升企业科技创新能力若干政策规定、促进科技和金融结合的实施意见，制定了科技“小巨人”企业培育、产业技术研究院建设、天使投资引导基金管理等配套政策。筹资2亿元，设立了创新创业风险投资基金。市级安排自主创新配套资金8.5亿元。新增省级以上研发机构48个，其中院士工作站8家、博士后科研工作站7家，安得物流技术中心获批国家级企业技术中心。国家级留学人员创业园挂牌。国家特种电线电缆产品质检中心、省级机械通用零部件产品质检中心、汽车电子及关键零部件创业园、江北科技企业孵化器一期基本建成。新增高新技术企业58家、创新型（试点）企业17家，分别占全省的11%和15%。高新技术产业增加值占地区生产总值比重提高1个百分点。发明专利申请量5300件，专利授权量9000件，分别占全省的18%、20%；万人有效发明专利拥有量6.7件。有效注册商标突破1万件。我市第六次蝉联全国科技进步先进市，繁昌和无为、芜湖县分别成为国家知识产权强县工程示范县、试点县。

（五）深化各项改革。着力深化重点领域改革，增强发展动力和活力。开展了营业税改征增值税试点扩围。出台了市级预算管理办法。准金融机构监管征信子系统中心平台开发建设基本完成。实施了模拟征迁试点，调整了土地和房屋征收安置办法，鼓励货币化补偿和现房安置，切实维护被征迁人合法权益。出台了市区商品房预售资金监管办法，促进房地产市场持续健康发展。组建了皖江城市建设投资公司，搭建城镇化建设融资平台。深化医药卫生体制改革，市县16家公立医院继续实施药品“零加成”销售，并在全国率先扩大到高值医用耗材，直接让利1.1亿元；推进了全科医生执业方式和服务模式改革试点；80%以上的社区卫生服务机构建立了全科医生团队，新增2家国家级示范社区卫生服务中心。计划生育工作跨入全省先进行列。居住证制度全面实施。出台了规范社会组织管理的意见。国家级社区服务规范化体系建设试点启动。社会服务管理信息化建设取得突破。我市列为首批国家智慧城市试点、国家信息消费试点、新一轮国家新能源汽车推广应用试点，卫生、地理、城市污水管理等信息资源实行整合共享。制定了节约集约用地政策措施，开发复垦整理新增耕地2.1万亩，盘活工业用地7041亩，建成标准化厂房275万平方米。出台了支持民营经济发展的55条意见，新登记企业5247户、个体工商户1.6万户，市场主体突破14万户。

（六）扩大对外开放。着力打造沿江开放高地，高质量承接产业转移。调整了招商引资目标管理考核和奖励办法，开展专业招商、精准招商、借会招商、项目招商。实际利用内资2276亿元、外资16.2亿美元，新增2家境外世界500强企业来芜投资。中欧生物科技产业园、信义电子材料科技产业园、三花节能环保产业园、奈伦城市综合体、健坤国际城等重大项目签约。新增12家企业赴境外投资。长江大桥开发区调整区位、新芜经济开发区和南陵工业园区扩区获批。编制了芜马城市组群城镇体系规划。凤鸣湖路等城市道路与马鞍山市实现互通，两市银行本票实现互兑。我市成为长三角城市经济协调会会员城市。亳州芜湖现代产业园区累计落户项目47个。三山港区设立了海关临时监管区，朱家桥外贸码头二期工程建设加快，芜湖港成为国家服务标准化试点单位，芜湖口岸成为全国运行管理先进口岸。

（七）提升城市品质。加强城市规划建设，统筹城乡发展。新一轮城市总体规划、土地利用总体规划获批。编制了综合交通体系、轨道交通线网、市域给水工程、商业网点等一批专项规划方案和规划建筑设计导则。皖赣铁路扩能改造芜宣段（商合杭铁路共线段）、芜湖火车站改扩建、弋江路快速化改造、弋江桥改造等项目开工建设。宁安城际铁路、铜南宣高速、芜申运河等工程加快推进。弋江路北延线立交、峨山路东延立交、205国道改造示范、206省道改建、中江大道南延、中山北路改造、沧津桥等工程基本建成。赤铸山路下穿工程、利民路东延段、长江南路通车。调增国省道461公里，实现了县县有国道。雕塑公园三期建成开放，汀棠、大阳垾、芦花荡、莲花湖等公园加快建设，新增绿地450万平方米，建成绿道30公里；完成森林增长工程17.7万亩，建成森林长廊91公里。完成了22座小型水库除险加固、7条中小河流治理项目，解决了17万农村人口饮水安全问题。我市列入全国水生态文明城市建设试点。编制了中心村规划导则。整合美好乡村资金4.8亿元，27个重点示范村建设基本完成。8880户农村危房改造全部完工。实施了节能减排项目103个。淘汰水泥落后产能24万吨，关闭矿山23家。4个污水处理厂竣工，完成35个老旧小区雨污分流。信义光伏发电项目竣工，严桥风电场开工建设。开展了空气质量新标准自动监测。单位生产总值能耗下降3.5%，化学需氧量、二氧化硫排放实现零增长，氨氮、氮氧化物排放分别下降2.3%和4.5%。建立了数字化城管视频监控系统，完善信息化、网格化管理，我市在全国城市文明程度指数测评中位居全省创建城市第一。

（八）发展各项社会事业。坚持经济建设与社会事业协调发展，不断改善民生。实施40项民生工程，完成工程投资80亿元。超额完成了学前教育三年行动计划。完成了71所学校标准化建设和农村小学教学点改造。三山中学新校区开工，工业学校、无为一中新校区等工程投入使用。启动中小学“名师工作室”建设。探索“名校+新校”的办学模式，安师大附外城东分校顺利开办。安徽工程大学机电学院新芜校区开工建设。安徽电子技术职业教育集团成立。实施全国教育信息化试点，“在线课堂”取得良好成效。实施创业富民工程，开展“科技创业年”活动，新增发放小额担保贷款13亿元，居全省首位。出台了21条优惠政策，扶持高校毕业生创新创业。完成就业技能培训1.7万人，培养高级工4800人、技师1300人。城镇登记失业率控制在3.5%以内。城镇职工养老、医疗、失业、工伤、生育保险参保总数达219万人次，增长3.7%。城乡居民养老保险参保163万人，48万人享受养老待遇。城镇居民医疗保险参保95万人，新型农村合作医疗参保228万人，基本实现全覆盖。完善大病医疗补充保险制度和医疗救助费用“一站式”结算办法，大病患者报销比例提升6个百分点。提高了居民最低生活保障标准，发放低保金5亿元，保障低保对象15.5万人。支出供养资金5470万元，保障五保供养对象1.4万人。建成7所敬老院和35个社区日间照料中心。向1.3万户困难群众发放临时生活救助资金2400万元。住房公积金政策实现全覆盖。基本建成保障房2.5万套，改造棚户区1.3万户。市博物馆、规划馆基本建成。天主堂等5个单位新增为全国重点文物保护单位。百年历史建筑复活计划一期项目完成。无为县、南陵县图书馆新馆获评国家一级馆。成功举办市第十二届运动会。启动第一次国有可移动文物普查、第三次经济普查。国防动员、双拥优抚、兵役工作进一步加强。档案、地方志、民族、宗教、文学艺术、气象、外事、侨务、对台、妇女儿童、科普、老龄、残疾人等事业取得新进步。“六五”普法持续推进，“律师进社区”实现全覆盖。开展开门大接访、干部下基层等活动，信访形势进一步好转。强化食品药品安全监管，实现网上公示制度全覆盖。安全生产死亡人数进一步下降。建立了金融、房地产及企业经营风险应对处置机制，基层应急处置的“南陵经验”在全省推广，社会治安稳定向好。

（九）加强政府自身建设。着力转变政府职能，提高推动科学发展、促进社会和谐的能力。出台了市政府工作规则，坚持科学民主决策，规范决策执行，健全监督制度。制定了公务员行为规范实施细则，提升服务水平。开展市级行政审批项目清理，取消、调整232项。实施了行政审批流程再造，完善了审批电子监察系统，提高网上受理率和办事效率。推进国家级行政管理与公共服务标准化试点，3个试点单位均通过中期评估。建立了电子化政府采购系统，政府采购实现网上全流程操作。加强了政府负债管理，严控行政支出，严禁盲目铺摊子，压减过于超前的基建投资。完善应急管理体系，启动了应急场所建设。制定了新闻发布和新闻发言人制度，公开政务信息8万条。办理人大议案2件、建议203件和政协提案351件。办理回复网民咨询和市长信箱留言1.2万条。严格执行中央和省、市转变工作作风相关规定，深入开展机关联系基层、干部联系群众、民主评议科长、政风行风评议等活动，政风建设进一步加强。推进惩治和预防腐败体系建设及廉政风险防控工作，强化审计监督，对腐败分子坚决处理。

一年来的实践探索，我们对推动芜湖发展的体会更加深刻：第一，坚持产业强市不放松。产业是立市之本、发展之基。必须把招商引资、产业培育、项目建设摆在更加突出的位置，面向全球配置资源，围绕首位产业、战略性新兴产业、新经济增长点等领域招大引强、招高引新，以大项目、大产业带动经济转型升级。第二，坚持提升城市品质不停顿。城市是产业发展和要素集聚的载体。只有高品质的城市，才能吸引高素质的人才、培育高水平的产业。必须树立城乡一体、组团发展、资源整合的理念，构建综合交通体系，提升教育、医疗、文化水平，打造宜居宜业宜游环境，提升城市能级。第三，坚持改革创新不畏难。创新是城市之魂，改革是不竭动力。必须打破惯性思维和自满心态，最大力度弘扬创新文化，最大范围释放改革红利，持续推进体制机制和制度创新，深入实施创新驱动发展战略，把解放思想、转变观念的成果转化为赢得新一轮区域竞争的强大动力。第四，坚持改善民生不止步。促进社会公平正义、增进人民福祉，是政府一切工作的出发点和落脚点。必须实施好民生工程，解决好群众最关心最直接最现实的利益问题，努力增加城乡居民收入，为社会提供多样化服务，让发展成果更多更公平惠及全体市民。第五，坚持可持续发展不动摇。既要立足当前，更要着眼长远；既要发展好经济，更要保护好环境，才能保持城市持久活力和竞争力。必须坚持一任接着一任干，一张蓝图绘到底，多做打基础、管长远的工作，努力创造经得起实践、历史、群众检验的实实在在的业绩。第六，坚持真抓实干不懈怠。实干兴市，空谈误事。推动科学发展，靠的是全市上下尤其是各级干部能干事、敢担当、善落实。实现宏伟目标，我们必须进一步增强忧患意识、责任意识，一时一刻不放松，一件一件抓落实，不容推诿扯皮，不容丝毫懈怠。

各位代表，去年的成绩来之不易。这是中共芜湖市委统揽全局、坚强领导的结果，是人大代表充分行使职权、政协委员积极参政议政的结果，是全市人民奋力进取、各界人士同心同德、广大干部苦干实干的结果。在此，我谨代表市人民政府，向各级人大代表、政协委员，向广大工人、农民、知识分子、干部、驻芜解放军指战员、武警官兵和政法干警，向各民主党派、工商联、各人民团体、无党派人士、离退休老同志和社会各界人士，向在芜湖的投资者、建设者，向关心支持芜湖发展的上级部门、驻芜单位和海内外友好人士，表示崇高的敬意和衷心的感谢！

我们也清醒地看到前进道路上还有不少困难，工作中还有一些不足，主要是：全市综合实力还不够强，经济外向度不高；产业结构还不够优，服务业发展不足；经济发展与资源环境的矛盾仍然突出，节能减排任务艰巨；城市对人才、资本、技术等要素集聚能力还不够强，外在形象与内在品质亟待提升；公共服务与群众期待还有较大差距，部分群众生活困难；各项管理制度有待完善，政府效能有待提升，为民服务需要改进。受宏观环境、市场需求等多重影响，去年我市地区生产总值增幅尽管高于全国、领先全省，但未能实现更好预期。对这些问题，我们一定高度重视，着力加以解决。

二、奋力开创改革发展新局面

党的十八届三中全会开启了新一轮改革大幕，作出了一系列重大制度安排，在我国现代化建设进程中具有里程碑意义，必将释放出巨大的改革红利。芜湖区位、产业、政策、生态等方面优势突出，正处于可以大有作为的黄金发展期。我们必须准确把握改革动向，积极抢抓宏观环境变化带来的重大机遇，着眼改革发展重点领域和关键环节，超前谋划创新之举、应对之策，抢占新一轮区域竞争、产业升级制高点，集聚发展新能量，打造竞争新优势，不断提升芜湖在长江经济带乃至全国的地位和影响力。

今年工作的总体要求是：全面贯彻党的十八大、十八届三中全会和中央、省经济工作会议精神，按照稳中求进、改革创新的总要求，坚持提高发展质量与扩张经济总量并举，更加注重要素集聚和产业升级，更加注重深化改革和扩大开放，更加注重城市功能完善和品质提升，更加注重资源节约和生态文明，更加注重民生改善和社会和谐，勇当全省乃至中部地区非省会城市发展的“领跑者”。

综合考虑各种因素，今年经济社会发展的主要预期目标是：在提高质量和效益的基础上，地区生产总值比上年增长11.5%，居民人均收入、财政收入同步增长，城镇新增就业7万人，节能减排完成省政府下达任务。重点做好以下工作：

（一）以跨江发展为重点，加快建设现代化大城市

坚持规划引领。加强城市发展战略和规划研究，深化江北新区功能定位、产业布局与城市形象设计。完成综合交通枢纽规划编制，加快城市轨道交通、城市排水防涝、公交、内河岸线等专项规划编制。开展重点地段城市设计工作。认真落实规划建筑设计导则，出台市政、园林设计和施工导则。按照主城区定位规划建设江北新区、三山区，启动无为撤县设市工作，按照中等城市标准推进南陵建设，加快芜湖县、繁昌县融入市区。

全面拉开江北建设框架。统筹开发江北新区，按照芜马城市组群中心区的标准加快建设沈巷片区，启动二坝、汤沟片区新型城镇化示范区建设。增加过江通道，开工建设商合杭铁路芜湖长江公铁大桥，加快合福铁路、长江公路二桥等工程进度。构建江北骨干交通网，开工建设庐铜铁路、江北大道、江北纬八路、通江大道北延线及互通立交等工程，加快建设北沿江高速公路、和谐大道。推进宝能组团等重点开发地块建设。在项目、融资、用地等方面，积极支持无为经济开发区城东园区、高沟经济开发区加快建设。

优化提升江南城区建设。实行棚户区改造、保障房建设、房屋征收、商品房供给有效联动。结合改造176万平方米棚户区，推进老城区有序更新，改善人居环境。推进住房公积金贷款支持保障房建设试点。启动建设神山口商圈，打造若干特色街区。完善城东新区商贸、教育、卫生等配套功能。推进生态城区建设，提升城市道路和街头绿化水平，建成神山公园、汀棠公园综合改造一期，新增绿地面积400万平方米，新造林19.5万亩。实施城市出入口综合整治。优化公交线路，增加出租车运力。推进市区高层住宅供水一体化，启动备用水源建设，整治地下管网。

加快建设城乡基础设施。完善立体交通网，力争芜宣机场早日开工，加快宁安城际铁路、皖赣铁路扩能改造、芜申运河等工程进度，完成火车站东站房改扩建、宁安城际铁路弋江站工程，加快国省道一级公路改造。优化老城区交通，开工建设中江桥改建、城南客运枢纽、黄山中路拓宽、钱桥立交等工程，完成弋江路北延线一期、九华路北延、仓津路、弋江桥改建、荆山桥改建工程，建成9座行人过街通道，建设一批社会公共停车场。开工建设裕溪河治理工程，加快青弋江分洪道、无为县万亩圩口达标、荆方排涝站、澛港闸（桥）等水利工程建设，确保城乡安全度汛。科学有序开展美好乡村建设。

提升城市管理水平。规范房地产开发行为，推进住宅产业化，强化商品房预售资金监管，促进房地产市场健康发展。制定城市夜景亮化总体规划及管理办法，规范优化户外广告设置。规范摊点管理，综合整治市容市貌，创新渣土车管理方式。推广建筑节能，倡导绿色建筑。开展重点区域环境整治行动，继续实施农村清洁工程。完善城市管理考核办法，发挥数字化城管平台作用。巩固提升文明创建成果，全力争创全国文明城市。

（二）以改革开放为动力，构筑体制机制新优势

大力推进重要领域和关键环节改革。完成地方政府机构改革。深化市政、园林等事业单位改革。深化招标采购制度改革，做好顶层设计，健全制度体系，提高科学性、公平性，有效堵塞漏洞。深化财税体制改革，积极研究事权与财权相匹配的财政管理制度，落实好市管县财政体制；调整优化财政专项支出，加强对财政资金使用情况的审计监督和绩效评价；清理、优化财政扶持发展的各项政策；推进税源专业化管理和营业税改征增值税扩围改革；探索政府购买社会组织服务机制。着力防控债务风险，制定加强政府性债务管理的办法，加强国有资产和政府融资平台的监督管理，规范举债审批程序，从源头上控制负债；各级财政建立偿债准备金，健全债务风险预警机制，加强债务问责。深化金融改革，支持大江银行等民营金融机构的申报设立；推动股权融资和发债工作，鼓励企业加快改制和境内外上市步伐，扩大“新三板”上市规模；构建市级平台牵头、覆盖县区的中小企业融资、投资、担保体系；筹建住宅政策性金融机构；积极利用保险资金支持基础设施建设；推进政府资产证券化，盘活资产；扩大联合征信系统覆盖面，完善地方金融监管体系，优化金融生态，防范金融风险。深化土地管理改革，做好土地开发复垦整理、城乡建设用地增减挂钩、工矿废弃地复垦利用等工作，推进国家级开发区存量建设用地二次开发利用试点工作，争取开展农村土地使用制度综合改革试点。完善土地使用税征管工作，坚决纠正工业、住宅用地改变规划用途行为。探索教育领域综合改革，推进城区学前教育集团化办学，增加优质教育资源供给；加快高中阶段学校结构性调整，探索综合高中办学模式；推进职业教育改革创新，深化产教合作，组建职教集团；支持在芜高校与国外大学、知名企业合作办学。深化医药卫生体制改革，加大公立医院改革力度，完善医疗服务基层首诊和双向转诊机制；推进医疗保险付费制度改革；鼓励社会办医，支持社会资本参与公立医院改制重组，积极引进高端医疗资源；完善基层医疗体制机制，提升基层医疗服务水平；推广全科医生制度。深化户籍制度改革，加快推行居住证制度，让进城农民享受城市居民的公共服务。

构建开放型经济体制。改进招商方式方法，围绕首位产业、新兴产业、服务业、龙头企业和产业链核心环节，强化精准招商、专业招商、以商招商，做好跟踪服务，推动项目落地。大力引进和培育外向型企业，支持现有开发区扩区升级。提升出口加工区，申报综合保税区，谋划自由贸易区，健全长三角区域大通关协作机制，建成雨耕山公用型保税仓库，抓好国家汽车及零部件出口基地建设。加快三山港区建设步伐，争取芜湖港吞吐量突破1亿吨，打造皖江航运枢纽。支持奇瑞、海螺等企业“走出去”，鼓励与国外企业合资合作。深化区域合作，加强与上海自由贸易试验区对接，推进芜湖港与上海港战略合作，把芜湖港建设成为上海港的主要喂给港；完善与长三角城市联动发展机制，积极参与南京都市圈活动；加快芜马同城化，推动芜亳合作共建。

（三）以产业和项目为根本，奋力打造经济升级版

大力发展民营经济。加强经济运行分析，落实好稳增长措施，坚决兑现降税减负和奖补政策，促进经济持续健康较快发展。支持民间资本进入基础设施、社会服务等领域，鼓励发展民营资本控股的混合所有制企业，推动民营经济大发展、大提升。建立基于信息化、覆盖全市的中小企业呼叫中心，实现服务企业机制化、常态化。培育一批“专精特新”中小企业和“小巨人”企业。

新上一批重大项目。全力引进和培育一批优质、高端的重大项目，支撑经济快速发展和产业扩张升级。坚持政府工作项目化、项目推进责任化、责任落实奖惩化，加大项目落实和推进力度。开工建设海创总部基地、钻石飞机及配套装备制造、科创生命健康产业城、古城一期等项目。加快埃夫特万台工业机器人及关键零部件等项目建设。建成中国非物质文化博览园、雨耕山文化创意产业园、星隆国际城、朱家桥外贸码头二期等项目。做好无为新材料产业园前期工作。推动繁昌循环经济产业园项目落地。

集聚发展首位产业和新兴产业。坚定不移把汽车及装备制造产业作为首位产业重点突破，支持奇瑞、海螺等龙头企业加快发展。推进企业重组和产业链重塑，增强龙头企业和产业竞争力。实施重大经济增长点培育工程，每年安排不少于10亿元资金，撬动500亿元项目投资，积极培育千亿元产业集群。明确各县区、开发区首位产业，建设产业关联度高、配套性强的产业集聚区。抓好机器人、新型显示2个国家级战略性新兴产业区域集聚发展试点，打造具有国际竞争力的自主品牌机器人产业。深入实施“千百亿”企业培育工程、战略性新兴产业“6122”工程。

大力发展服务业。以服务业集聚区建设为载体，加快发展研发设计、现代物流、检验检测、信息服务等生产性服务业，积极发展健康养老、文化旅游、体育健身等生活性服务业。制定培育服务业大企业大集团的政策措施。支持镜湖区争创国家服务业综合改革试点区。推进公铁水联运，加快建设现代物流中心。支持三只松鼠等电商核心企业发展，建设电子商务集聚区，创建国家电子商务示范城市。加快“智慧芜湖”建设，启动政务云计算中心、“天网工程”等项目，完善人口、法人、房产等基础数据库，加快“一户一企一网页”建设。围绕欢乐城市目标，加快重大旅游项目建设，提升旅游行业整体服务水平。办好第六届中国（芜湖）科普产品博览交易会。

积极发展现代农业。完成都市型现代农业、现代林业发展规划修编。加快建设无为羽毛羽绒、繁昌食品、南陵食品3个省级农业产业园区。推动龙头企业、家庭农场、合作社、种养大户等经营主体加快发展。引导优质工商资本投向现代农业，加快信义光伏发电与生态农业一体化等项目建设。深入开展地方种质资源保护，打造大米、江蟹等农产品品牌，办好芜湖农博会。支持奇瑞重工做大做强，打造国内外知名的农业装备产业集群。

（四）以建设国家创新型城市为目标，提升经济核心竞争力

大规模集聚创新资源。落实科技创新政策措施，推动国家创新型城市、知识产权示范市、文化和科技融合示范基地建设。更加突出招才引智，用活人才特区政策，实施人才引进培育、创新创业、平台建设、服务提升工程，面向全球导入优秀人才、创新团队。优化创新平台，推进国家级留学人员创业园、院士工作站、博士后科研工作站、技能大师工作室建设。推动企业与知名高校、科研院所开展交流合作。完善人才服务体系，营造全社会尊重人才、各类人才广泛集聚、人才资源效益充分发挥的生动局面。

进一步激发创新活力。支持企业创建省级以上研发机构、产业技术研究院。支持企业申报高新技术企业、创新型企业、高新技术产品。鼓励发明创造，提高专利质量和专利成果转化率。实施科技“小巨人”企业培育计划，重点培育80家科技“小巨人”企业。新增300家“智慧企业”。加快建设科技企业孵化器，建立有效的孵化机制和管理体制。深化企业股权和分红激励试点、专利权质押贷款试点，完善科技融资担保等金融中介服务，拓宽科技型、创新型中小企业融资渠道。

推动资源节约和环境保护。加强节能预警监测，控制能源消费。实施严桥风电等项目。加大新能源汽车推广应用力度，扶持新能源汽车产业发展。引导水泥、电线电缆、汽车零部件、矿山等行业实施并购重组，推动资源向优势企业集中。推行土地管理信息化，实现“一张图”管理。健全用地指标与项目质量挂钩机制，开展县区单位生产总值建设用地耗地率、开发区节约集约用地评价，争取绿色矿业示范区建设试点，争创国土资源节约集约模范市。推动开发区循环化改造，支持经济技术开发区创建国家生态工业示范区，支持繁昌争创国家级循环经济示范县。实施老城区雨污分流，开工建设城南生活垃圾焚烧发电厂，建成餐厨废弃物资源化利用和无害化处理工程。完善污染物排放许可制度，建立重点减排项目和重要行业产量月调度、季督查制度。加强饮用水源地监管，实现水源保护全覆盖。落实大气污染综合整治行动计划，实施机动车污染治理等工程。创建国家环保模范城市。

（五）以保障改善民生为着力点，推进和谐社会建设

计划投入83.5亿元，实施33项民生工程。

做好就业和社会保障工作。健全政府促进就业责任制度，加强就业技能培训，推进就业援助，结合产业升级开发更多优质岗位，帮扶高校毕业生、农村转移劳动力、城镇就业困难人员就业。加强创业服务载体建设，完善社区创业服务工作平台，加快形成创业带动就业良性机制。鼓励和引导青年、妇女创业就业。扶持科技创业实体，活跃高科技创业氛围。完善社会保障体系，给广大群众以稳定、可靠的民生保障预期。研究城镇职工养老保险与城乡居民养老保险制度衔接办法。完善大病医疗补充保险制度，推进城镇职工基本医疗保险、城镇居民医疗保险和生育保险市级统筹，实行医疗保险省内异地就医结算。农村低保人均补差标准提高10%。落实社会救助和保障标准与物价上涨挂钩联动机制。加强基层救助能力建设，提升自然灾害救助水平。建设居家养老服务体系，引进社会力量兴办养老护理院。

打造教育名城和皖江医疗中心。优化教育资源配置，促进教育公平公正。通过名校办分校、委托式办学等方式，办好新建学校。开工建设宝能国际双语学校。依托在芜高校基础教育资源优势，培育一批名教师、名校长、名学校。保障中小学生每天锻炼一小时，切实提高学生身体素质。启动芜湖职业技术学院升本工作。完成市一院新区儿童医学中心、皖南康复医院病房大楼项目，开工建设宝能、和天等民营医院，建设专科医疗服务和公共卫生集聚区。保持新型农村合作医疗参合率在98%以上。加快建立统一高效、互联互通、信息共享的区域卫生协同服务模式，提高基本公共卫生服务质量。落实国家生育政策，提高出生人口素质，促进人口计生工作转型发展。

协调发展各项社会事业。推进公共文化设施建设，开放市博物馆、规划馆，争创国家级档案馆，构建现代公共文化服务体系。支持国有文化企业改革发展，鼓励非公有制文化企业做大做强，促进文化大发展大繁荣。办好中国体育旅游博览会。深化供销社管理体制改革。推进“六五”普法、“律师进社区、进乡村”、法律援助活动。建成气象科普中心，启动交通气象、城市气象灾害监测预警工程。完成第三次全国经济普查、第六次全国体育场地普查。做好民族、宗教、外事、侨务、对台、妇女儿童、残疾人等工作，发展档案、地方志、人防、防灾减灾等事业。完成全国城市少数民族流动人口服务管理体系建设试点。积极推动军民融合深度发展，增强全民国防意识，支持驻芜部队建设，落实优抚安置政策，开展双拥共建活动。

创新社会治理体制。加强基层政权建设，做好第九届村委会换届工作。深化社区管理体制改革，做实做强社区，推行社区网格化管理，提升社区服务、管理能力。完善社区工作者激励保障机制。实施政社分开，完善社会组织培育、扶持政策。高标准推进社会服务管理信息化建设。强化市场价格监管，稳定价格总水平。严厉打击侵犯知识产权和制售假冒伪劣商品行为。建立最严格的覆盖全过程的食品药品监管制度，落实企业主体责任。推进肉菜流通追溯体系建设。创新社会矛盾纠纷化解机制，实行网上受理信访等制度，依法规范信访秩序。完善安全生产监管模式，健全道路交通等隐患排查治理长效机制。加强防灾减灾救灾工作，提高应急保障能力。加强社会治理，加大依法管理网络力度，严密防范和惩治各类违法犯罪活动。

三、努力建设人民满意的政府

面对新形势新任务新要求，我们必须以开展群众路线教育实践活动为契机，强化政府自身建设，提高政府工作科学化水平，加快建设法治政府、创新政府、服务政府、廉洁政府，增强政府的公信力和执行力。

（一）严格依法行政，建设法治政府。落实法治国家、法治政府、法治社会一体建设的要求，运用法治思维和法治方式提高行政能力。严格执行市政府工作规则，依法用权履职。认真执行市人大及其常委会的决议和决定，重大决策出台前向人大报告。自觉接受人大监督和政协监督。建立健全协商制度，广泛听取各民主党派、工商联、无党派人士和工会、共青团、妇联等人民团体的意见。高度重视社会公众和舆论监督。严格规范性文件制定程序，创建一批依法行政示范单位。推进行政执法规范化。完善政务和办事公开制度，推进决策公开、管理公开、服务公开、结果公开，及时主动公开涉及公众利益的信息。

（二）转变政府职能，建设创新政府。正确处理政府与市场、与社会的关系，以行政审批制度改革为突破口，简政放权，全面正确履行政府职能。探索实行政府及工作部门权力清单制度，减少对微观经济干预。强化公共服务、市场监管、社会治理、环境保护等职责，更好发挥政府作用。加强与国内外知名咨询机构合作，完善重大行政决策咨询机制。规范政府合同管理，严格履约监管。完善发展成果考核评价体系。

（三）坚持高效便捷，建设服务政府。强化公仆意识和服务理念，带头践行开放、诚信、务实、创新的城市精神，落实公务员行为规范实施细则。健全“机关联系基层、干部联系群众”长效机制，在政府部门、服务窗口、基层站所推行“亲切服务”，寓管理于服务、帮扶之中。优化工作流程，改进服务方式，打造更好发展环境，提供更优公共服务。引入现代管理制度，坚决整治“庸懒散”，调动每个工作人员积极性。发挥市民心声网等平台的桥梁纽带作用，强化政民互动，办理并及时回复群众的合理意见和建议。继续开展民主评议百名科长、万人千企评议政风行风等活动。创新政府工作人员考核办法，开发网上考评系统。

（四）强化制度约束，建设廉洁政府。加强对转变工作作风相关规定落实情况的监督检查，深入推进惩治和预防腐败体系建设，严格落实廉政责任制。健全各项制度，用制度管权管事管人，加强对主要领导干部行使权力的制约和监督，把权力关进制度的笼子里。贯彻党政机关厉行节约反对浪费条例，做到财政供养人员、“三公经费”只减不增，政府性楼堂馆所一律停建，一般性支出预算压减5%。强化廉政风险防控，加大监察和审计监督力度，严肃查处违纪违规行为。

各位代表！芜湖发展正处于蓄势突破的关键阶段。我们既要登高望远，精谋良策，更要扑下身子，苦干实干。让我们在中共芜湖市委的坚强领导下，团结拼搏，奋勇争先，谱写好中国梦的芜湖篇章，在建设创新、优美、和谐、幸福新芜湖的征程中迈出更加坚实的步伐！
